# 2-[2-(2,4-Dinitro­phen­yl)eth­yl]-1,3,5-trinitro­benzene

**DOI:** 10.1107/S1600536811041031

**Published:** 2011-10-08

**Authors:** Zhi-Hua Wei, Wen-Yan Wang, Ying Diao, Jian-Long Wang

**Affiliations:** a School of Chemical Engineering and Environment, North University of China, Taiyuan, People’s Republic of China

## Abstract

In the title compound, C_14_H_9_N_5_O_10_, the two benzene rings are inclined at a dihedral angle of 14.81 (5)°, and the nitro groups are twisted with respect to the benzene rings to which they are attached, making dihedral angles of 57.89 (7), 14.93 (7), 62.58 (7), 2.80 (12) and 22.38 (12)°. Weak inter­molecular C—H⋯O hydrogen bonding is present in the crystal structure.

## Related literature

The title compound is an inter­mediate in the synthesis of the high energy density compound 2,2′,4,4′,6,6′-hexa­nitro­stilbene, see: Shipp (1964 ▶). For the synthesis, see: Blatt & Rytina (1950 ▶).
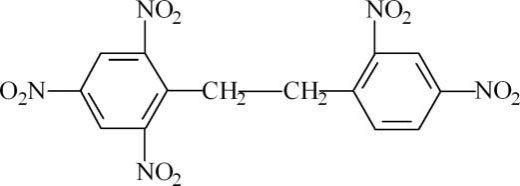


## Experimental

### 

#### Crystal data

C_14_H_9_N_5_O_10_
*M* *_r_* = 407.26
Monoclinic, 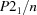

*a* = 14.099 (7) Å
*b* = 8.227 (4) Å
*c* = 15.356 (8) Å
β = 114.758 (7)°
*V* = 1617.6 (14) Å^3^
*Z* = 4
Mo *K*α radiation
μ = 0.15 mm^−1^
*T* = 113 K
0.20 × 0.18 × 0.12 mm

#### Data collection

Rigaku Saturn724 CCD diffractometer
Absorption correction: multi-scan (*CrystalClear*; Rigaku/MSC, 2000 ▶) *T*_min_ = 0.971, *T*_max_ = 0.983
16454 measured reflections
3823 independent reflections
2847 reflections with *I* > 2σ(*I*)
*R*_int_ = 0.043

#### Refinement

*R*[*F* ^2^ > 2σ(*F* ^2^)] = 0.040
*wR*(*F* ^2^) = 0.107
*S* = 1.03
3823 reflections
262 parameters
H-atom parameters constrained
Δρ_max_ = 0.35 e Å^−3^
Δρ_min_ = −0.33 e Å^−3^

### 

Data collection: *CrystalClear* (Rigaku/MSC, 2000 ▶); cell refinement: *CrystalClear*; data reduction: *CrystalClear*; program(s) used to solve structure: *SHELXTL* (Sheldrick, 2008 ▶); program(s) used to refine structure: *SHELXTL*; molecular graphics: *SHELXTL*; software used to prepare material for publication: *SHELXTL*.

## Supplementary Material

Crystal structure: contains datablock(s) I, global. DOI: 10.1107/S1600536811041031/xu5347sup1.cif

Structure factors: contains datablock(s) I. DOI: 10.1107/S1600536811041031/xu5347Isup2.hkl

Supplementary material file. DOI: 10.1107/S1600536811041031/xu5347Isup3.cml

Additional supplementary materials:  crystallographic information; 3D view; checkCIF report

## Figures and Tables

**Table 1:** Hydrogen-bond geometry (Å, °)

*D*—H⋯*A*	*D*—H	H⋯*A*	*D*⋯*A*	*D*—H⋯*A*
C4—H4⋯O8^i^	0.95	2.39	3.249 (2)	151
C10—H10⋯O2^ii^	0.95	2.58	3.508 (3)	167
C11—H11⋯O9^ii^	0.95	2.40	3.353 (3)	176

Symmetry codes: (i) 
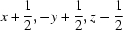
; (ii) 
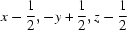
.

**Fig. 1.**
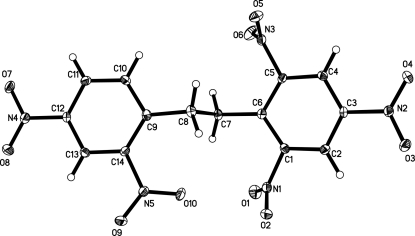
The molecular structure of the title compound with the atom numbering scheme. Displacement ellipsoids are drawn at the 30% probability level.

**Fig. 2.**
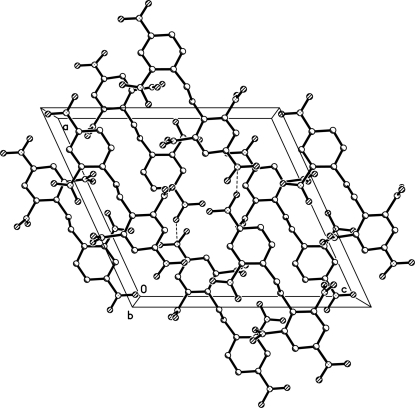
The crystal packing of the title compound.

## supplementary crystallographic information

### Comment

2,2',4,4',6,6'-Hexantanitrobibenzyl, which can be prepared from bibenzyl by nitration (Blatt & Rytina, 1950), is a intermediate for synthesizing high energy density compound 2,2',4,4',6,6'-hexanitrostilbene (Shipp, 1964). As a byproduct, 2,2',4,4',6–pentantanitrobibenzyl is separated from the nitrate product. Here we report the crystal structure of the title compound.

In the crystal structure, because the number of nitro group is not identical in two benzene rings, the two benzene rings are inclined at a dihedral angle 14.811 (48)°, For the interaction of nitro groups, the nitro groups is rotated out the benzene plane, making dihedral angles of 57.885 (65)°(N1/O1, O2), 14.934 (68)°(N2/O3, O4), 62.579 (71)° (N3/O5, O6), 2.799 (121)°(N4/O7, O8) and 22.376 (115)° (N5/O9, O10).

### Experimental

The title compound was prepared according to literature method (Blatt *et al.*, 1950). Single crystals were obtained by evaporation of a solution of the title compound in ethyl acetate at room temperature.

### Refinement

All the Friedel pairs were merged. All H atoms were positioned geometrically and treated as riding, with C—H bond lengths constrained to 0.95 ° for benzene ring H and 0.99 ° for methylene H atoms, and with *U*_iso_(H) = 1.2*U*_eq_(C).

### Crystal data

**Table:**

C_14_H_9_N_5_O_10_	*F*(000) = 832
*M**_r_* = 407.26	*D*_x_ = 1.672 Mg m^−^^3^
Monoclinic, *P*2_1_/*n*	Mo *K*α radiation, λ = 0.71073 Å
Hall symbol: -P 2yn	Cell parameters from 5515 reflections
*a* = 14.099 (7) Å	θ = 1.6–27.9°
*b* = 8.227 (4) Å	µ = 0.15 mm^−^^1^
*c* = 15.356 (8) Å	*T* = 113 K
β = 114.758 (7)°	Prism, colorless
*V* = 1617.6 (14) Å^3^	0.20 × 0.18 × 0.12 mm
*Z* = 4	

### Data collection

**Table:**

Rigaku Saturn724 CCD diffractometer	3823 independent reflections
Radiation source: rotating anode	2847 reflections with *I* > 2σ(*I*)
multilayer	*R*_int_ = 0.043
Detector resolution: 14.22 pixels mm^-1^	θ_max_ = 27.9°, θ_min_ = 1.7°
ω and φ scans	*h* = −18→12
Absorption correction: multi-scan (*CrystalClear*; Rigaku/MSC, 2000)	*k* = −10→10
*T*_min_ = 0.971, *T*_max_ = 0.983	*l* = −20→19
16454 measured reflections	

### Refinement

**Table:**

Refinement on *F*^2^	Primary atom site location: structure-invariant direct methods
Least-squares matrix: full	Secondary atom site location: difference Fourier map
*R*[*F*^2^ > 2σ(*F*^2^)] = 0.040	Hydrogen site location: inferred from neighbouring sites
*wR*(*F*^2^) = 0.107	H-atom parameters constrained
*S* = 1.03	*w* = 1/[σ^2^(*F*_o_^2^) + (0.0594*P*)^2^] where *P* = (*F*_o_^2^ + 2*F*_c_^2^)/3
3823 reflections	(Δ/σ)_max_ = 0.002
262 parameters	Δρ_max_ = 0.35 e Å^−^^3^
0 restraints	Δρ_min_ = −0.33 e Å^−^^3^

### Special details

**Table:**

Geometry. All e.s.d.'s (except the e.s.d. in the dihedral angle between two l.s. planes) are estimated using the full covariance matrix. The cell e.s.d.'s are taken into account individually in the estimation of e.s.d.'s in distances, angles and torsion angles; correlations between e.s.d.'s in cell parameters are only used when they are defined by crystal symmetry. An approximate (isotropic) treatment of cell e.s.d.'s is used for estimating e.s.d.'s involving l.s. planes.
Refinement. Refinement of *F*^2^ against ALL reflections. The weighted *R*-factor *wR* and goodness of fit *S* are based on *F*^2^, conventional *R*-factors *R* are based on *F*, with *F* set to zero for negative *F*^2^. The threshold expression of *F*^2^ > σ(*F*^2^) is used only for calculating *R*-factors(gt) *etc*. and is not relevant to the choice of reflections for refinement. *R*-factors based on *F*^2^ are statistically about twice as large as those based on *F*, and *R*- factors based on ALL data will be even larger.

### Fractional atomic coordinates and isotropic or equivalent isotropic displacement parameters (Å^2^)

**Table:**

	*x*	*y*	*z*	*U*_iso_*/*U*_eq_	
O1	0.64474 (9)	0.08191 (14)	1.03105 (8)	0.0325 (3)	
O2	0.68105 (8)	0.33883 (14)	1.06099 (7)	0.0270 (3)	
O3	0.86607 (8)	0.48825 (14)	0.85862 (7)	0.0273 (3)	
O4	0.79057 (8)	0.43068 (14)	0.70657 (7)	0.0283 (3)	
O5	0.39540 (8)	0.33811 (14)	0.60692 (8)	0.0291 (3)	
O6	0.40895 (9)	0.07989 (14)	0.63732 (8)	0.0369 (3)	
O7	−0.06620 (8)	0.15196 (13)	0.84028 (8)	0.0264 (3)	
O8	0.02060 (9)	0.19441 (17)	0.99188 (8)	0.0408 (3)	
O9	0.37441 (8)	0.36062 (14)	1.11500 (7)	0.0281 (3)	
O10	0.46932 (9)	0.29102 (18)	1.04226 (9)	0.0450 (4)	
N1	0.65521 (9)	0.22023 (16)	1.00726 (9)	0.0217 (3)	
N2	0.79545 (9)	0.42757 (15)	0.78811 (8)	0.0199 (3)	
N3	0.43956 (9)	0.21995 (16)	0.65550 (8)	0.0204 (3)	
N4	0.01476 (10)	0.18383 (16)	0.91063 (9)	0.0224 (3)	
N5	0.38449 (10)	0.31254 (16)	1.04428 (9)	0.0227 (3)	
C1	0.63893 (11)	0.24823 (17)	0.90684 (10)	0.0171 (3)	
C2	0.72280 (11)	0.31584 (17)	0.89544 (10)	0.0186 (3)	
H2	0.7866	0.3392	0.9490	0.022*	
C3	0.71003 (11)	0.34782 (17)	0.80322 (10)	0.0163 (3)	
C4	0.61836 (11)	0.31584 (17)	0.72466 (10)	0.0174 (3)	
H4	0.6110	0.3381	0.6614	0.021*	
C5	0.53752 (11)	0.25001 (17)	0.74139 (10)	0.0166 (3)	
C6	0.54234 (11)	0.21345 (17)	0.83201 (10)	0.0168 (3)	
C7	0.44685 (11)	0.16298 (17)	0.84700 (11)	0.0193 (3)	
H7A	0.4687	0.1085	0.9099	0.023*	
H7B	0.4046	0.0853	0.7964	0.023*	
C8	0.38085 (11)	0.31511 (19)	0.84336 (11)	0.0224 (3)	
H8A	0.4257	0.3962	0.8901	0.027*	
H8B	0.3553	0.3642	0.7787	0.027*	
C9	0.28820 (11)	0.27651 (17)	0.86572 (10)	0.0184 (3)	
C10	0.19285 (11)	0.24056 (18)	0.78975 (10)	0.0206 (3)	
H10	0.1892	0.2383	0.7266	0.025*	
C11	0.10317 (11)	0.20795 (18)	0.80267 (10)	0.0207 (3)	
H11	0.0392	0.1831	0.7497	0.025*	
C12	0.10966 (10)	0.21278 (17)	0.89524 (10)	0.0168 (3)	
C13	0.20092 (11)	0.24750 (17)	0.97324 (10)	0.0184 (3)	
H13	0.2037	0.2512	1.0361	0.022*	
C14	0.28871 (11)	0.27693 (17)	0.95696 (10)	0.0174 (3)	

### Atomic displacement parameters (Å^2^)

**Table:**

	*U*^11^	*U*^22^	*U*^33^	*U*^12^	*U*^13^	*U*^23^
O1	0.0369 (7)	0.0277 (7)	0.0361 (7)	0.0037 (5)	0.0186 (5)	0.0142 (5)
O2	0.0267 (6)	0.0344 (7)	0.0198 (6)	−0.0049 (5)	0.0096 (5)	−0.0043 (5)
O3	0.0223 (6)	0.0302 (7)	0.0264 (6)	−0.0099 (5)	0.0074 (5)	−0.0005 (5)
O4	0.0273 (6)	0.0392 (7)	0.0234 (6)	−0.0053 (5)	0.0156 (5)	0.0023 (5)
O5	0.0249 (6)	0.0293 (7)	0.0249 (6)	0.0011 (5)	0.0023 (5)	0.0041 (5)
O6	0.0293 (7)	0.0235 (7)	0.0455 (7)	−0.0058 (5)	0.0035 (6)	−0.0108 (5)
O7	0.0151 (6)	0.0305 (6)	0.0307 (6)	−0.0029 (4)	0.0069 (5)	−0.0059 (5)
O8	0.0273 (7)	0.0792 (10)	0.0204 (6)	−0.0048 (6)	0.0143 (5)	0.0012 (6)
O9	0.0260 (6)	0.0374 (7)	0.0180 (5)	0.0030 (5)	0.0063 (5)	−0.0062 (5)
O10	0.0147 (6)	0.0825 (11)	0.0368 (7)	−0.0038 (6)	0.0097 (5)	−0.0248 (7)
N1	0.0182 (7)	0.0254 (7)	0.0224 (7)	0.0017 (5)	0.0094 (5)	0.0051 (5)
N2	0.0185 (7)	0.0211 (7)	0.0211 (7)	−0.0016 (5)	0.0094 (5)	0.0018 (5)
N3	0.0172 (6)	0.0243 (7)	0.0216 (6)	−0.0020 (5)	0.0100 (5)	−0.0048 (5)
N4	0.0180 (7)	0.0263 (7)	0.0227 (7)	−0.0004 (5)	0.0083 (6)	0.0007 (5)
N5	0.0170 (7)	0.0268 (7)	0.0228 (7)	−0.0007 (5)	0.0069 (5)	−0.0055 (5)
C1	0.0193 (7)	0.0166 (7)	0.0172 (7)	0.0013 (5)	0.0094 (6)	0.0021 (5)
C2	0.0168 (7)	0.0186 (7)	0.0185 (7)	0.0007 (6)	0.0056 (6)	0.0011 (6)
C3	0.0144 (7)	0.0162 (7)	0.0213 (7)	−0.0004 (5)	0.0103 (6)	0.0005 (5)
C4	0.0195 (7)	0.0178 (7)	0.0158 (7)	0.0017 (6)	0.0083 (6)	−0.0005 (5)
C5	0.0127 (7)	0.0178 (7)	0.0183 (7)	0.0007 (5)	0.0053 (6)	−0.0032 (5)
C6	0.0173 (7)	0.0136 (7)	0.0203 (7)	0.0006 (5)	0.0089 (6)	−0.0012 (5)
C7	0.0179 (8)	0.0194 (8)	0.0242 (8)	−0.0028 (6)	0.0122 (6)	−0.0015 (6)
C8	0.0221 (8)	0.0217 (8)	0.0269 (8)	−0.0003 (6)	0.0137 (7)	0.0000 (6)
C9	0.0190 (8)	0.0160 (7)	0.0223 (8)	0.0013 (5)	0.0107 (6)	0.0001 (6)
C10	0.0229 (8)	0.0247 (8)	0.0163 (7)	0.0015 (6)	0.0101 (6)	−0.0007 (6)
C11	0.0181 (8)	0.0239 (8)	0.0171 (7)	0.0005 (6)	0.0044 (6)	−0.0027 (6)
C12	0.0148 (7)	0.0176 (7)	0.0199 (7)	0.0003 (5)	0.0091 (6)	0.0000 (6)
C13	0.0203 (8)	0.0194 (7)	0.0164 (7)	0.0015 (6)	0.0084 (6)	0.0003 (5)
C14	0.0138 (7)	0.0172 (7)	0.0186 (7)	0.0004 (5)	0.0041 (6)	−0.0024 (6)

### Geometric parameters (Å, °)

**Table:**

O1—N1	1.2229 (17)	C4—C5	1.380 (2)
O2—N1	1.2305 (17)	C4—H4	0.9500
O3—N2	1.2289 (15)	C5—C6	1.397 (2)
O4—N2	1.2252 (16)	C6—C7	1.516 (2)
O5—N3	1.2248 (17)	C7—C8	1.547 (2)
O6—N3	1.2211 (17)	C7—H7A	0.9900
O7—N4	1.2272 (16)	C7—H7B	0.9900
O8—N4	1.2187 (18)	C8—C9	1.517 (2)
O9—N5	1.2187 (16)	C8—H8A	0.9900
O10—N5	1.2222 (17)	C8—H8B	0.9900
N1—C1	1.4789 (19)	C9—C10	1.394 (2)
N2—C3	1.4727 (18)	C9—C14	1.398 (2)
N3—C5	1.4774 (18)	C10—C11	1.386 (2)
N4—C12	1.472 (2)	C10—H10	0.9500
N5—C14	1.4801 (19)	C11—C12	1.386 (2)
C1—C2	1.383 (2)	C11—H11	0.9500
C1—C6	1.395 (2)	C12—C13	1.372 (2)
C2—C3	1.376 (2)	C13—C14	1.383 (2)
C2—H2	0.9500	C13—H13	0.9500
C3—C4	1.374 (2)		
			
O1—N1—O2	125.21 (13)	C5—C6—C7	122.30 (12)
O1—N1—C1	118.10 (13)	C6—C7—C8	109.39 (12)
O2—N1—C1	116.66 (12)	C6—C7—H7A	109.8
O4—N2—O3	124.75 (12)	C8—C7—H7A	109.8
O4—N2—C3	118.08 (12)	C6—C7—H7B	109.8
O3—N2—C3	117.16 (12)	C8—C7—H7B	109.8
O6—N3—O5	124.67 (13)	H7A—C7—H7B	108.2
O6—N3—C5	118.09 (12)	C9—C8—C7	112.61 (12)
O5—N3—C5	117.23 (12)	C9—C8—H8A	109.1
O8—N4—O7	123.76 (13)	C7—C8—H8A	109.1
O8—N4—C12	118.46 (12)	C9—C8—H8B	109.1
O7—N4—C12	117.78 (12)	C7—C8—H8B	109.1
O9—N5—O10	123.38 (13)	H8A—C8—H8B	107.8
O9—N5—C14	117.97 (13)	C10—C9—C14	115.96 (13)
O10—N5—C14	118.65 (13)	C10—C9—C8	118.37 (13)
C2—C1—C6	124.74 (13)	C14—C9—C8	125.65 (13)
C2—C1—N1	115.28 (12)	C11—C10—C9	122.64 (14)
C6—C1—N1	119.91 (13)	C11—C10—H10	118.7
C3—C2—C1	117.19 (13)	C9—C10—H10	118.7
C3—C2—H2	121.4	C10—C11—C12	117.96 (13)
C1—C2—H2	121.4	C10—C11—H11	121.0
C4—C3—C2	122.44 (13)	C12—C11—H11	121.0
C4—C3—N2	118.51 (13)	C13—C12—C11	122.44 (13)
C2—C3—N2	118.96 (12)	C13—C12—N4	118.48 (13)
C3—C4—C5	117.27 (13)	C11—C12—N4	119.06 (12)
C3—C4—H4	121.4	C12—C13—C14	117.52 (13)
C5—C4—H4	121.4	C12—C13—H13	121.2
C4—C5—C6	124.78 (13)	C14—C13—H13	121.2
C4—C5—N3	115.83 (13)	C13—C14—C9	123.46 (13)
C6—C5—N3	119.38 (13)	C13—C14—N5	114.64 (13)
C1—C6—C5	113.57 (13)	C9—C14—N5	121.89 (13)
C1—C6—C7	123.63 (13)		
			
O1—N1—C1—C2	−122.42 (14)	N3—C5—C6—C7	−7.6 (2)
O2—N1—C1—C2	55.76 (17)	C1—C6—C7—C8	93.28 (16)
O1—N1—C1—C6	60.38 (18)	C5—C6—C7—C8	−78.03 (17)
O2—N1—C1—C6	−121.44 (15)	C6—C7—C8—C9	−175.40 (12)
C6—C1—C2—C3	−1.3 (2)	C7—C8—C9—C10	−92.16 (15)
N1—C1—C2—C3	−178.37 (12)	C7—C8—C9—C14	89.32 (18)
C1—C2—C3—C4	0.4 (2)	C14—C9—C10—C11	0.4 (2)
C1—C2—C3—N2	176.88 (12)	C8—C9—C10—C11	−178.27 (14)
O4—N2—C3—C4	−15.38 (19)	C9—C10—C11—C12	0.4 (2)
O3—N2—C3—C4	163.70 (13)	C10—C11—C12—C13	−0.3 (2)
O4—N2—C3—C2	167.96 (13)	C10—C11—C12—N4	178.04 (13)
O3—N2—C3—C2	−12.96 (19)	O8—N4—C12—C13	1.2 (2)
C2—C3—C4—C5	0.3 (2)	O7—N4—C12—C13	−179.59 (13)
N2—C3—C4—C5	−176.20 (12)	O8—N4—C12—C11	−177.28 (13)
C3—C4—C5—C6	−0.2 (2)	O7—N4—C12—C11	2.0 (2)
C3—C4—C5—N3	178.94 (12)	C11—C12—C13—C14	−0.6 (2)
O6—N3—C5—C4	117.21 (15)	N4—C12—C13—C14	−178.95 (12)
O5—N3—C5—C4	−62.02 (17)	C12—C13—C14—C9	1.5 (2)
O6—N3—C5—C6	−63.64 (18)	C12—C13—C14—N5	−179.64 (13)
O5—N3—C5—C6	117.13 (15)	C10—C9—C14—C13	−1.4 (2)
C2—C1—C6—C5	1.4 (2)	C8—C9—C14—C13	177.18 (14)
N1—C1—C6—C5	178.36 (12)	C10—C9—C14—N5	179.81 (13)
C2—C1—C6—C7	−170.56 (14)	C8—C9—C14—N5	−1.6 (2)
N1—C1—C6—C7	6.4 (2)	O9—N5—C14—C13	−21.49 (19)
C4—C5—C6—C1	−0.7 (2)	O10—N5—C14—C13	157.85 (15)
N3—C5—C6—C1	−179.73 (12)	O9—N5—C14—C9	157.42 (14)
C4—C5—C6—C7	171.45 (13)	O10—N5—C14—C9	−23.2 (2)

### Hydrogen-bond geometry (Å, °)

**Table:**

*D*—H···*A*	*D*—H	H···*A*	*D*···*A*	*D*—H···*A*
C4—H4···O8^i^	0.95	2.39	3.249 (2)	151
C10—H10···O2^ii^	0.95	2.58	3.508 (3)	167
C11—H11···O9^ii^	0.95	2.40	3.353 (3)	176

Symmetry codes: (i) *x*+1/2, −*y*+1/2, *z*−1/2; (ii) *x*−1/2, −*y*+1/2, *z*−1/2.
